# Hydroquinone, an Environmental Pollutant, Affects Cartilage Homeostasis through the Activation of the Aryl Hydrocarbon Receptor Pathway

**DOI:** 10.3390/cells12050690

**Published:** 2023-02-22

**Authors:** Cintia Scucuglia Heluany, Anna De Palma, Nicholas James Day, Sandra Helena Poliselli Farsky, Giovanna Nalesso

**Affiliations:** 1 Department of Comparative Biomedical Sciences, School of Veterinary Medicine, Faculty of Health and Medical Sciences, University of Surrey, Guildford GU2 7AL, UK; 2 Department of Clinical and Toxicological Analyses, School of Pharmaceutical Sciences, University of São Paulo, São Paulo 015508-000, Brazil

**Keywords:** environmental pollutant, smoking, chondrocyte, cartilage, oxidative stress, IL-1β

## Abstract

Exposure to environmental pollutants has a proven detrimental impact on different aspects of human health. Increasing evidence has linked pollution to the degeneration of tissues in the joints, although through vastly uncharacterised mechanisms. We have previously shown that exposure to hydroquinone (HQ), a benzene metabolite that can be found in motor fuels and cigarette smoke, exacerbates synovial hypertrophy and oxidative stress in the synovium. To further understand the impact of the pollutant on joint health, here we investigated the effect of HQ on the articular cartilage. HQ exposure aggravated cartilage damage in rats in which inflammatory arthritis was induced by injection of Collagen type II. Cell viability, cell phenotypic changes and oxidative stress were quantified in primary bovine articular chondrocytes exposed to HQ in the presence or absence of IL-1β. HQ stimulation downregulated phenotypic markers genes SOX-9 and Col2a1, whereas it upregulated the expression of the catabolic enzymes MMP-3 and ADAMTS5 at the mRNA level. HQ also reduced proteoglycan content and promoted oxidative stress alone and in synergy with IL-1β. Finally, we showed that HQ-degenerative effects were mediated by the activation of the Aryl Hydrocarbon Receptor. Together, our findings describe the harmful effects of HQ on articular cartilage health, providing novel evidence surrounding the toxic mechanisms of environmental pollutants underlying the onset of articular diseases.

## 1. Introduction

Exposure to environmental pollutants can severely affect and compromise human health. Hydroquinone (HQ), a benzene metabolite, is a widespread pollutant agent used in several commercial and industrial processes, such as dye intermediate, and as stabilizer in paints and motor fuels [1] and represents 3% of the particle matter phase of cigarette smoke [2]. HQ has a proven pro-carcinogenic activity and can mediate immune- and mielo-toxicity by promoting apoptosis and oxidative stress [3,4,5,6].

The effects of pollution on musculoskeletal health remain mostly uncharacterised. While some studies showed the potential danger of pollutants on chondrogenesis and joint formation [7,8], our knowledge of their effect and mechanism of action in adult tissues is very limited. We have recently demonstrated that exposure to HQ can aggravate joint damage in an experimental animal model of inflammatory arthritis: HQ promoted inflammation, an increased influx of immune cells, synovial proliferation and oxidative stress in mice and rats in which inflammatory arthritis was induced by subcutaneous injection of collagen type II (Collagen-induced arthritis model, CIA), through the activation of the Aryl Hydrocarbon Receptor (AhR) pathway [9,10,11]. Importantly, these studies showed a synergistic effect of HQ in exacerbating the degenerative effects of pro-inflammatory cytokines such as IL-1β and TNFα in pathological conditions in the joint [11]. However, the effect of HQ on articular cartilage (AC) has not been investigated yet. 

The AC is the connective tissue covering the edges of the bones in the diarthrodial joints. Articular chondrocytes are the only type of cells populating the tissue [12]. They are responsible for the synthesis and turnover of collagens and highly sulphated proteoglycans, which are the main components of the thick extracellular matrix (ECM), conferring the biomechanical properties upon the tissue [13,14]. The AC is elastic and resistant to compression and allows the joints to move smoothly [14]. 

Degeneration of the AC is a common feature in several musculoskeletal conditions, such as osteoarthritis (OA) and rheumatoid arthritis (RA), which affect millions of people worldwide and represent, therefore, a very heavy socio-economic burden. The characterisation of factors influencing their onset can help in designing strategies to prevent or delay their onset and reduce their impact on patients’ quality of life. 

In this work, we investigated the effect of HQ on articular chondrocytes to elucidate how it can affect articular cartilage health. We showed that proteoglycan content was significantly reduced in the articular cartilage of rats exposed to HQ in comparison to vehicle-exposed animals. HQ decreased the viability of isolated chondrocytes, promoted oxidative stress and downregulated the expression of phenotypic marker genes alone and in synergy to Interleukin-1β (IL-1 β), one of the main pro-inflammatory cytokines promoting cartilage degeneration both in inflammatory and non-inflammatory arthritis. HQ mediated its catabolic activity in articular chondrocytes through the activation of the AhR pathway. Together, our findings suggest that the AhR activation in articular chondrocytes could be an important mechanism through which pollutants can compromise cartilage health. 

## 2. Material and Methods

### 2.1. Animals and Collagen-Induced Arthritis (CIA)

Six to eight-week-old male Wistar rats were supplied by the Animal Facility of the Faculty of Pharmaceutical Sciences and Chemistry Institute of the University of São Paulo. Rats were supplemented with food and water ad libitum. All procedures were performed according to the guidelines of the Brazilian Society of Science of Laboratory animals for the proper care and use of experimental animals. Experimental procedures were approved by the Ethics Committee on Animal Use (CEUA) of the University of São Paulo (protocol number 435). Before euthanasia, animals were anaesthetised with a solution containing Ketamine/Xylazine (Ceva Sante Animale, Paulinea, Brazil) 80:8 mg/kg through intraperitoneal injection.

### 2.2. Collagen-Induced Arthritis (CIA) Induction and In Vivo HQ Exposure

CIA induction was performed as described before [15]. Two mg/mL of bovine collagen type II (Chondrex, Woodinville, WA, USA) was dissolved in 0.1 M acetic acid (LabSynth, Diadema, Brazil), by gentle stirring and the solution was emulsified in equal volumes of Complete Freund Adjuvant (CFA, Sigma-Aldrich, St. Louis, MO, USA). At day 7, 200 µL of the emulsified solution was injected subcutaneously at the base of the tail, and 100 µL was administered on day 14 through the same route. For comparison, the non-CIA group was used as a healthy control group.

CIA rats were divided into two groups: one group was exposed to an HQ solution (Hydroquinone 99%, Sigma-Aldrich, St. Louis, MO, USA) at 25 ppm (1.5 mg/60 mL) through an ultrasonic nebuliser (NS^®^, São Paulo, Brazil) for 1 h/daily for 35 consecutive days as previously described [9,10,11]; the control group was instead exposed to a vehicle solution (5% ethanol in saline) for the same amount of time.

### 2.3. Isolation and Culture of Bovine Articular Chondrocytes and Cartilage Explants 

Bovine articular cartilage explants were isolated from the metatarsal and metacarpal joints of adult cows purchased within less than 24 h of death from a local abattoir. As tissues and cells were removed from cows euthanised in an approved abattoir and were not killed for the purpose of this study, no ethical approval was deemed to be needed by the Ethical Committee of the University of Surrey. The articular cartilage was dissected in sterile conditions and processed for chondrocyte isolation or explant cultures as previously described [14]. Bovine articular chondrocytes (BACs) were cultured with complete medium (CM-DMEM F12 (Thermo Fisher, Waltham, MA, USA), supplemented with 10 % Fetal Bovine Serum (FBS; Gibco Invitrogen, Carlsbad, CA, USA) and 1% of antibiotics and antimycotic solution (Sigma-Aldrich, St. Louis, MO, USA) at 37 °C in a 5% CO_2_ incubator (Binder, Tuttlingen, Germany). Cells at passages 1 to 3 were used for the experiments described in this manuscript.

### 2.4. MTT Assay 

Cell viability was measured through the 3-(4,5-dimethylthiazol-2-yl)-2,5 diphenyltetrazolium bromide (MTT; Sigma-Aldrich, St. Louis, MO, USA) method. One hundred thousand BACs/well were seeded in 96-well plates and cultured in CM for 24 h. Thereafter, cells were washed with PBS and cultured with DMEM/F-12 supplemented with 0.1% of bovine serum albumin (BSA; Sigma-Aldrich, St. Louis, MO, USA) for 24 h. BACs were treated as described in the individual experiments. Then, 0.5 mg/mL of MTT solution was added to each well and incubated for 3 h in the dark at 37 °C. The medium was then removed, and the blue formazan crystals were dissolved in 200 µL of dimethyl sulfoxide (DMSO; Sigma-Aldrich, St. Louis, MO, USA). The optical density reading was recorded at 570 nm in a CLARIOstar plate reader (BMG LABTECH, Offenburg, Germany). 

### 2.5. FACS Assay

Cell viability and death were determined by a flow cytometry-based method using Annexin V (BD Biosciences, San Jose, CA, USA) and 7-Aminoactinomycin D (7AAD; BD Biosciences, San Jose, CA, USA) double-staining. BACs (1 × 10^4^ cells/well) were seeded in 24-well plates and cultured with CM overnight. Then, cells were washed with PBS and treated with HQ (10, 25 or 50 µM) for 24 h. After that, cells were washed with PBS, detached with trypsin (Gibco—Fisher Scientific, Pittsburgh, PA, USA), centrifuged and incubated with an APC-conjugated Annexin V (1:100) for 30 min at room temperature. The cells were then incubated with 7-AAD (1:200) and 10,000 events were recorded in a flow cytometer (Accuri C6, BD Biosciences, San Jose, CA, USA). Data are expressed as a percentage of viable cells (double negative population), apoptotic cells (APC positive and 7-AAD negative), necrotic cells (APC negative and 7-AAD positive) or late apoptotic cells (APC and 7-AAD positive).

### 2.6. Gene Expression

For gene expression analyses, 7.5 × 10^4^ BACs were plated per well in 24-well plates and cultured with CM for 24 h. The cells were washed with PBS and treated as described in the Results section. Total RNA was extracted from BACs using TRIzol reagent (Invitrogen, Carlsbad, CA, USA), according to the manufacturer’s instructions. Three hundred and fifty nanograms of total RNA from each sample were reverse transcribed to cDNA using a High-Capacity cDNA Reverse Transcription Kit (Applied Biosystems, Waltham, MA, USA). Quantitative PCR was performed with hot-start DNA polymerase (Qiagen, Hilden, Germany), as previously described [16]. Primer sequences are listed in Table 1 (All primers were purchased from Sigma-Aldrich, St. Louis, MO, USA). All data were normalised to internal control of β-actin values. All experiments were performed in a PCR system (CFX96TM Optics Module; BioRad, Hercules, CA, USA). 

### 2.7. Micromass Cultures 

BACs were plated at a density of 25 × 10^4^ cells per well in a complete medium in 24-well plates. Micromass cultures were prepared as described before [17]. The micromasses were stimulated as described in the individual experiments with HQ ± IL-1β (20 ng/mL; RP0106B-025 Kingfisher Biotech, Saint Paul, MN, USA). After 48 h, the culture media were collected, and the micromasses were washed with PBS and fixed with cold methanol (Sigma-Aldrich, St. Louis, MO, USA). 

### 2.8. Alcian Blue Staining 

Micromass cultures were stained overnight with Alcian blue (Carl Roth, Karlsruhe, Germany) as previously described [17]. Stained cultures were washed extensively with distilled water and were extracted with 6M guanidine hydrochloride (Sigma-Aldrich, St. Louis, MO, USA). The optical density of the extracted cultures was read at 630 nm with a CLARIOstar spectrophotometer (BMG LABTECH, Offenburg, Germany). The absorbance values were normalised to protein content determined by the Bicinchoninic acid assay (BCA) assay, following manufacturer instructions (ThermoFisher Scientific; Waltham, MA, USA). Images of the micromasses were acquired at room temperature with a stereomicroscope (SZTL 350 Stereo Binocular Microscope, VWR^®^; Radnor, PA, USA).

### 2.9. Safranin-O Staining 

Three-micrometre thick sagittal sections of bovine cartilage explants and 5 μm sections of metatarsal regions from CIA-rats paws were stained with safranin-O (SO; Sigma-Aldrich, St. Louis, MO, USA) as described before [16]. Images were taken using the same settings for all the images on an optical microscope (Eclipse Ci, Nikon, Tokyo, Japan). Images were acquired through 10× magnification objective lenses, and the SO-staining was quantified by using ImageJ (NIH; Bethesda, MD, USA). Four fields per slide (totalling 16 images per experimental group) were acquired, and the mean of the staining intensity of each group was compared.

For the paws, the histologic scoring system was evaluated and scored in a blinded manner by two investigators, following the Standardised Microscopic Arthritis Scoring of Histological section (SMASH) recommendations for scoring histological sections from inflammatory arthritis experimental animal models [18].

### 2.10. DMMB Assay 

The release of glycosaminoglycans (GAGs) by micromass cultures of BACs and bovine cartilage explants in culture media was measured by using the Dimethyl methylene Blue (DMMB, Sigma-Aldrich, St. Louis, MO, USA) assay (adapted from Farndale et al., 1982 [19]. Briefly, 200 µL of the DMMB solution was added to 20 µL of culture media in a 96-well plate. Subsequently, the absorbance was measured at 525 nm with a CLARIOstar spectrophotometer (BMG LABTECH, Offenburg, Germany). 

### 2.11. Quantification of Nitric Oxide by Griess Assay 

Fifty microliters of Sulphanilamide solution (1% in 5% phosphoric acid; Promega; Madison, WI, USA) were added to 50 µL of the culture media of micromass cultures of BACs or bovine cartilage explants treated with HQ ± IL-1β (20 ng/mL). After a 10 min incubation at RT, 50 µL of N-1-napthylenediamine dihydrochloride solution (NED, 0.1% in water; Promega, Madison, WI, USA) was added to the mix. The absorbance was then measured at 550 nm with a CLARIOstar spectrophotometer (BMG LABTECH, Offenburg, Germany).

### 2.12. Quantification of Reactive Oxygen Species by Dichloro-Dihydro-Fluorescein Diacetate (DCFH-DA) Assay

The intracellular accumulation of reactive oxygen species (ROS) was quantified in BACs cultures using the fluorescent probe CM-H2DCFDA (Invitrogen, Carlsbad, CA, USA). Ten thousand chondrocytes/well were seeded in 24-well plates and stimulated with HQ for 24 h. The cells were then incubated with 10 μM CM-H2DCFDA for 30 min at 37 °C in the dark. The cells were resuspended in PBS and 10,000 events were acquired in a MACSQuant flow cytometer (Miltenyi Biotec, Bergisch Gladbach, Germany). Results are presented as arbitrary units of fluorescence. 

### 2.13. Reporter Assay

BACs were seeded at a density of 7 × 10^4^ cells/well in CM in a 24-well plate. The cells were co-transfected with 450 ng/well of pGL4.43 [luc2P/XRE/Hygro] luciferase reporter vector (Promega, Madison, WI, USA) and with 50 ng/well of the control vector expressing *Renilla reniformis* luciferase by using Lipofectamine (ThermoFisher, Waltham, MA, USA) following manufacturer instructions. After transfection, the cells were stimulated with CM (vehicle) or with the AhR ligand 6-formylindolo(3,2b)carbazole (FICZ, 10 µM, Sigma-Aldrich, St. Louis, MO, USA) or with HQ (10 or 25 µM) for 24 h. Luciferase activity was determined using the Dual luciferase reporter assay system (Promega, Madison, WI, USA). Firefly luciferase activity was normalised by the *Renilla* luciferase activity. Data are expressed as a fold increase of relative luminescence units in comparison to the vehicle. 

### 2.14. Statistical Analysis 

One-way ANOVA with the Tukey-Post test was used to compare the statistical differences between multiple groups, and a two-tailed *t*-test was used for comparisons between two groups. Values of *p* < 0.05 were considered statistically significant. Data are expressed as the mean  ±  standard error of the mean (SEM). Statistical analyses were performed using GraphPad Prism version 8.0 (GraphPad Software, Boston, MA, USA). 

## 3. Results

### 3.1. Exposure to HQ Exacerbates Cartilage Damage in an In Vivo Model of Inflammatory Arthritis

**Figure 1:**
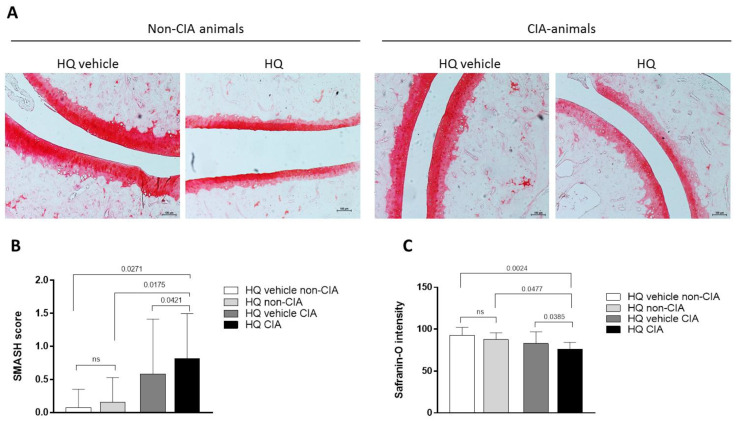
In vivo HQ exposure exacerbates cartilage damage in an experimental model of inflammatory arthritis. Exposure to HQ, by nebulisation, during 35 consecutive days, increased the loss of proteoglycan in the articular cartilage of the paw joints of CIA-exposed animals (**A**–**C**), quantified by using the SMASH scoring system. 10× magnification Data represented mean ± SEM from four independent experiments and were analysed by one-way ANOVA.

Rheumatoid arthritis (RA) is a chronic inflammatory musculoskeletal condition whose hallmarks are synovial inflammation, bone erosion and cartilage damage [20]. We previously showed that in CIA animals, exposure to HQ exacerbated synovial inflammation [9,10,11]. Here we show that while exposure to HQ did not significantly alter cartilage structure and composition in healthy rats, HQ increased proteoglycan loss in the articular cartilage of the paw joints of the treated CIA animals in comparison to control ones (Figure 1A–C). This suggests a more widespread toxic effect of HQ on joint tissues in pathological conditions.

### 3.2. Low Concentrations of HQ Slow Down Chondrocyte Growth without Compromising Cell Viability

To investigate the effects of HQ exposure on chondrocyte homeostasis, we treated bovine articular chondrocytes with different concentrations of HQ (1 μM up to 100 μM) and monitored cell growth across time by using an MTT assay.

**Figure 2:**
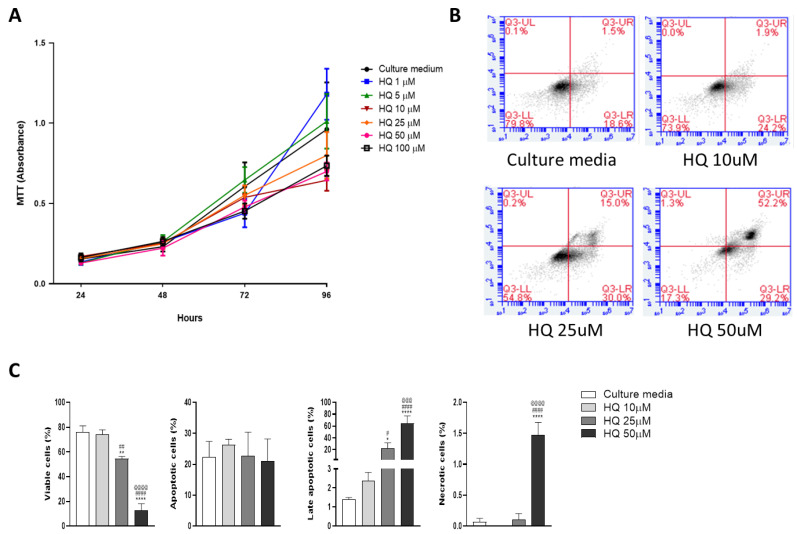
In vitro HQ exposure reduces viability and promotes apoptosis in a time and concentration-dependent manner. Stimulation of BACs with increasing concentrations of HQ (1–100 µM) halted cell growth in a time-dependent fashion as evaluated by MTT assay (**A**). HQ treatment promoted cell death at the protein level upon exposure to a concentration of 50 µM after 24 h of treatments (**B**,**C**), as quantified by the FACs method using Annexin V and 7-AAD dye. Data represented mean ± SEM from three independent experiments and were analysed by one-way ANOVA. (**A**): 24H: *p* = 0.0215 CM vs. 1 µM; *p* = 0.0220 CM vs. 5 µM; *p* = 0.0010 CM vs. 10 µM; *p* = 0.014 CM vs. 25 µM; *p* = 0.0001 CM vs. 50 and 100 µM. 72H: *p* = 0.0049 CM vs. 25 µM; *p* = 0.0001 CM vs.50 and 100 µM. 96H: *p* = 0.0262 CM vs. 10 µM; *p* = 0.0001 CM vs. 25, 50 and 100 µM. (**C**): *, **, **** vs. Culture media; #, ##, #### vs. HQ 10 µM; @@@, @@@@ vs. HQ 25 µM.

Ten to one hundred μM HQ slowed cell growth over a 96h time course (Figure 2A). Apoptosis was promoted by the xenobiotic at concentrations higher than 25 μM, as shown by increased Annexin V expression measured by FACS analysis (Figure 2B,C).

### 3.3. HQ Modulates the Expression of Phenotypic Markers in Articular Chondrocytes

We then tested the effect of HQ on the expression of cartilage phenotypic markers and matrix remodelling enzymes, as their modulations are hallmarks of the initiation of pro-catabolic events leading to cartilage degradation in different forms of arthritis [21,22].

**Figure 3:**
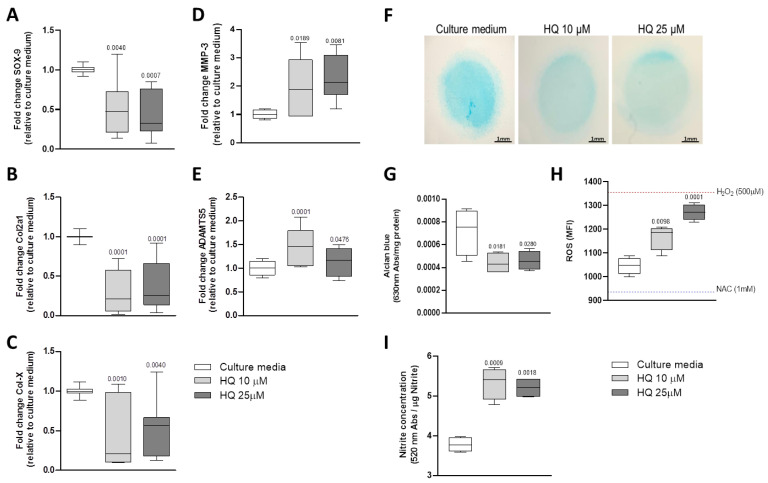
HQ exposure modulates the expression of phenotypic markers in articular chondrocytes. HQ stimulation of monolayer cultures of BACs downregulated the phenotypic markers SOX-9 (**A**), Col2a1 (**B**), and Col-X (**C**) but upregulated the catabolic enzymes MMP-3 (**D**) and ADAMTS5 (**E**). HQ exposure reduced the production of highly sulphated GAGs (**F**,**G**) over 48 h incubation on BACs in micromass cultures, as measured by Alcian Blue quantification. HQ also increased ROS generation (**H**), quantified by DCFH-DA assay and nitrite production (**I**), quantified by Griess reaction in micromass cultures. qPCR results were normalised to the housekeeping gene β-actin and expressed as fold change in comparison to culture media. MFI = median of fluorescence intensity. Data represent mean ± SEM from three independent experiments and were analysed with one-way ANOVA.

Incubation of BACs with 10 μM and 25 μM of HQ promoted downregulation of SRY-box transcription factor 9 (SOX-9), collagen type II (Col2a1) and collagen type X (Col-X) mRNAs (Figure 3A–C) whilst it upregulated the expression of the matrix remodelling enzymes metalloprotease 3 (MMP-3) and A disintegrin and metalloproteinase with thrombospondin motifs 5 (ADAMTS5) mRNAs (Figure 3D,E).

Proteoglycan content was overall reduced in chondrocyte micromasses exposed to HQ (Figure 3F,G). 

Our results, therefore, confirm a pro-catabolic activity of HQ in the articular cartilage.

### 3.4. HQ Triggers Oxidative Stress in Articular Chondrocytes

HQ can induce the generation of ROS in different biological systems [10,11,23]. Increased oxidative stress in chondrocytes has been strongly associated with cartilage degeneration in musculoskeletal conditions [24]. We then tested whether HQ exposure could influence the production of ROS in BACs cultures. Our data showed that incubation of chondrocytes with HQ for 24 h was sufficient to trigger a significant increase of ROS in treated cells in comparison to non-stimulated cells (Figure 3H).

Nitric oxide (NO) metabolism can also suppress the synthesis of proteoglycans (PGs) and stimulate MMPs activity [25]. Stimulation of articular chondrocytes with HQ for 48 h significantly increased nitrite production, as evaluated by the Griess reaction (Figure 3I). 

### 3.5. HQ Enhanced the Pro-Catabolic Activity of IL-1β 

**Figure 4:**
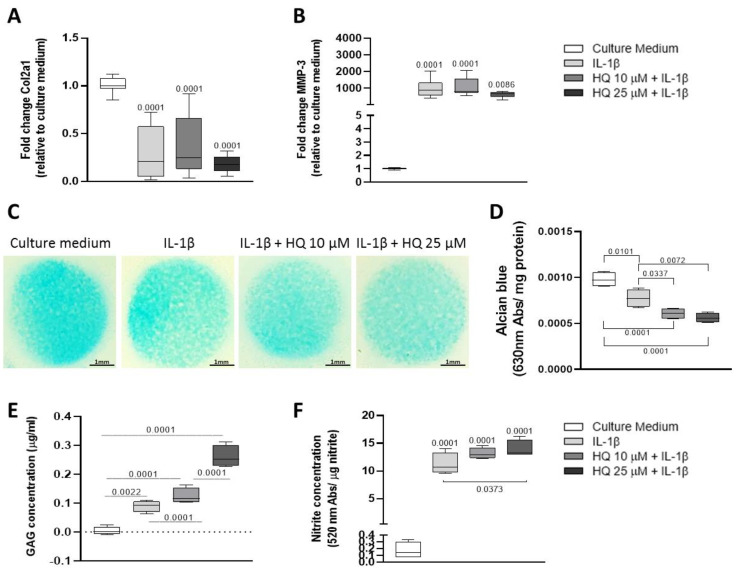
HQ treatment enhances the pro-catabolic activity of IL-1β in articular chondrocytes. Pre-incubation of monolayer cultures of BACS with IL-1β (20 ng/mL) for 4 h, did not further decrease the expression of the phenotypic marker Col2a1 (**A**) and of the catabolic enzyme MMP-3 mRNAs in cells stimulated with HQ (10 or 25 µM) for 48 h (**B**). The co-treatment, however, reduced the accumulation of highly sulphated GAGs in chondrocyte micromasses (**C**,**D**) and enhanced the release of GAGs (**E**), as assessed respectively by Alcian Blue staining and DMMB assay. IL-1β and HQ also had a combined effect in promoting nitrite production (**F**), as measured by the Griess reaction. qPCR results were normalised to the housekeeping gene β-actin and expressed as fold change to culture media. Data represent mean ± SEM from three independent experiments and were analysed with one-way ANOVA.

**Figure 5:**
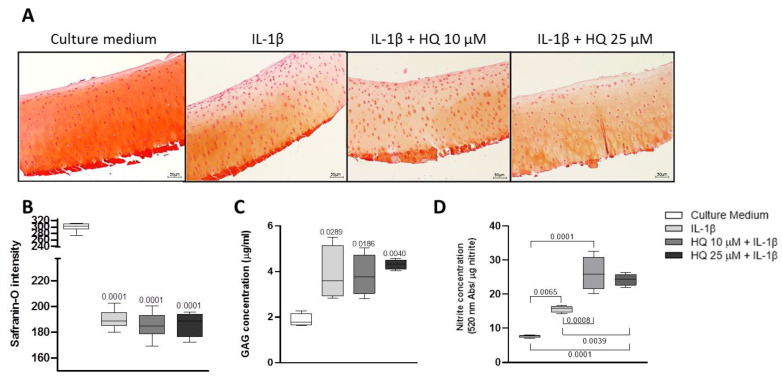
HQ treatment increases the pro-inflammatory activity of IL-1β in articular cartilage explants. Bovine articular cartilage explants were pre-incubated with CM or with IL-1β (20 ng/mL) for 4 h and subsequently incubated with HQ (10, 25 or 50 µM) for 72 h. The co-stimulation did not alter GAGs synthesis and release (**A**–**C**) but increased nitrite production (**D**) in comparison to stimulation with IL-1β alone. 10× magnification. Data represent ± SEM from four independent experiments and were analysed with one-way ANOVA.

Interleukin 1beta (IL-1β) is a pro-inflammatory cytokine overexpressed in the articular cartilage during the progression of both osteoarthritis and rheumatoid arthritis [26,27,28]. Thus, to investigate whether HQ could exacerbate the pro-inflammatory effect of this cytokine, BACs were pre-stimulated with a recombinant bovine IL-1β at 20 ng/mL for 4 h before being exposed to different concentrations of HQ for 24 or 48 h. HQ did not exacerbate the pro-catabolic effects of IL-1β in modulating the expression of phenotypic markers and matrix remodelling enzymes in monolayer cultures of BACs (Figure 4A,B). However, HQ stimulation further reduced the content and increased the release of GAGs in micromass cultures of BACs (Figure 4C–E). While this synergic effect was not reproduced in cultures of bovine cartilage explants (Figure 5A–C), HQ further increased nitrite production induced by IL-1β in both culture systems (Figure 4F and Figure 5D). 

### 3.6. HQ Activates the AhR Pathway in Articular Chondrocytes 

**Figure 6:**
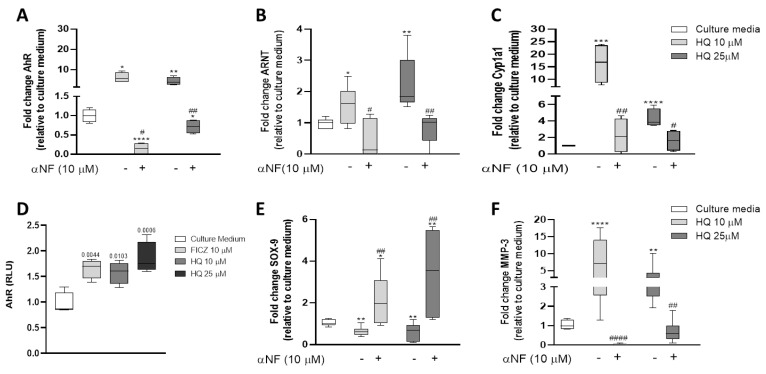
HQ activates the AhR pathway in articular chondrocytes: Modulation of components of the AhR pathway was determined by measuring mRNA levels of AhR, ARNT and Cyp1a1 in BACs incubated with CM or with HQ (10, 25 or 50 µM) in the absence or presence of αNF (10 µM) for 24 h (**A**–**C**). Results were normalised to the housekeeping gene β-actin and are expressed as fold change to CM. (**D**) Activation of the AhR receptor by HQ was confirmed through a luciferase-based reporter assay. Inhibition of AhR with αNF rescued the downregulation of SOX-9 (**E**) and the upregulation of MMP-3 (**F**) mediated by HQ. Data represent mean ± SEM from three independent experiments and were analysed by one-way ANOVA. * *p* < 0.05, ** *p* < 0.01, *** *p* < 0.001 and **** *p* < 0.0001 vs. CM; # *p* < 0.05, ## *p* < 0.01 and #### *p* < 0.0001 vs. αNF.

We previously demonstrated that HQ mediates its cytotoxicity in the synovium via activation of the AhR pathway in murine experimental models of inflammatory arthritis [9,10,11]. To test whether this mechanism of action is preserved in the articular chondrocytes, we measured the expression levels of AhR, of the aryl hydrocarbon receptor nuclear translocator (ARNT) and of the Cytochrome P450 Family 1 Subfamily A Member 1 (Cyp1a1), an endpoint target gene in the AhR pathway in BACS stimulated with 10 μM and 25 μM HQ. All the genes were upregulated in stimulated chondrocytes (Figure 6A–C). This upregulation was rescued by pre-incubation of the cells with α-naphthoflavone (αNF, 10 µM; Sigma-Aldrich, St. Louis, MO, USA), an AhR antagonist (Figure 6A–C). 

In its activated form, the AhR translocates from the cytoplasm to the nucleus and forms a heterodimer with the ARNT, which binds to specific DNA sequences located in the xenobiotic responsive elements (XRE) present in the promoter regions of the target genes (50-TA/TGCGTG-30) genes, regulating their expression [29,30]. 

To demonstrate the activation of the AhR pathway, we, therefore, performed a luciferase-based XRE reporter assay in articular chondrocytes stimulated for 24 h with HQ. As shown in Figure 6D, treatment with HQ increased the reporter gene activity in stimulated cells in a concentration-dependent manner. Finally, we showed that both the phenotypic changes as well as the pro-catabolic activity induced by HQ were mediated via the activation of the AhR pathway, as the HQ-mediated downregulation of SOX-9 and upregulation of MMP-3 were rescued by the pre-incubation of the articular chondrocytes with the AhR antagonist αNF (Figure 6E,F). 

## 4. Discussion

Inflammatory arthritis and osteoarthritis are diseases, respectively, affecting ~0.30% of the worldwide population [31] and 16% of the population over the age of 15 [32]. Both diseases are characterised by the degeneration of joint tissues. Several factors can contribute to the onset of these diseases and have been largely described. Nonetheless, the impact of environmental pollution on pre-existing musculoskeletal conditions is still vastly unknown. 

Here we focused on investigating whether HQ, a benzene metabolite contained in motor fuels and other environmental pollutants, can exacerbate the degenerative effect of inflammation on the articular cartilage.

Exposure to HQ decreased chondrocyte viability and promoted apoptosis in a dose and time-dependent manner, confirming previous observations in different biological systems [5,6]. 

In musculoskeletal conditions such as osteoarthritis and rheumatoid arthritis, the articular chondrocytes undergo phenotypic changes: the expression of phenotypic markers is altered, and the activity of matrix remodelling enzymes increase [21,33,34]. These changes contribute to the progressive loss of the biomechanical properties of the tissue and ultimately lead to its degradation [35,36]. Our data suggest that HQ could heavily contribute to many of these phenomena. 

Here we showed that exposure to HQ increased cartilage damage in the paws of rats in which inflammatory arthritis was induced by injection of Collagen type II. To better understand the molecular mechanisms modulated by HQ in the tissue, we stimulated isolated cells to the xenobiotic: HQ promoted downregulation of Col2a1 and SOX-9 while also inducing upregulation of MMP-3, which can degrade several types of collagens, proteoglycans and matrix proteins in the articular cartilage [37]. The pro-catabolic activity induced by HQ was also shown by the reduction of GAG staining and increased GAG release upon exposure of chondrocytes micromass cultures to the xenobiotic.

Oxidative stress can be induced through several mechanisms and can contribute to cellular metabolic decline as well as promote degenerative mechanisms [38]. The induction of oxidative stress in chondrocytes has been strongly associated with increased cartilage degradation in musculoskeletal conditions [23]. Moreover, nitrite production has also been shown to contribute to oxidative stress and degeneration of tissue integrity in the joints [39]. Previous data showed that exposure to environmental pollutants could promote ROS and NO generation in chondrocytes [40]. HQ exposure has been associated with oxidative damage and could affect the oxidative balance in other biological systems [9,10,11,41,42]. Indeed, here we confirmed the pro-oxidative effect of HQ on the articular chondrocytes, which could contribute to the overall phenotypic changes induced by the xenobiotic on these cells. 

Interleukin 1β (IL-1β) is a major pro-inflammatory trigger in both osteoarthritis and rheumatoid arthritis. IL-1 stimulates catabolic changes, suppresses anabolic pathways and decreases matrix synthesis [27]. We previously demonstrated the synergistic effect of HQ and inflammation in promoting synovial tissue degeneration [9,10,11]. Here we wanted to investigate whether we could see the same detrimental effect on the articular cartilage to determine whether environmental pollution could be considered a contributing factor in the degradation of this tissue in pathological conditions. Indeed IL-1β and HQ had a synergistic effect in reducing proteoglycan content and in promoting oxidative stress in articular chondrocytes, suggesting that exposure to environmental pollutants could potentiate inflammatory processes involved in the degradation of the articular cartilage. Interestingly, similar effects were observed in synoviocytes derived from patients affected by rheumatoid arthritis and co-stimulated with HQ and TNF-alpha [11]. 

AhR is a ligand-dependent transcription factor that translocates to the nucleus upon activation by xenobiotics and pollutants [43]. In this study, we showed that the HQ mediates its toxic effects through the activation of the AhR pathway. We demonstrated that HQ upregulated the expression of the AhR itself and of its downstream effectors, suggesting that xenobiotics and pollutants can directly influence the health of the articular cartilage. 

We confirmed that after HQ exposure, AhR translocates from cytoplasm to the nucleus, forms a heterodimer with the AhR nuclear translocator (ARNT) and promotes the transcription of target genes such as Cyp1a1. The activation of the AhR pathway has been shown to mediate several detrimental effects, such as aggravation of articular diseases, cancer development and endocrine disruption [44,45,46]. Indeed, we and others previously showed that this receptor plays a major role in exacerbating rheumatoid arthritis in smokers [9,10,11,46,47,48].

Interestingly our data showed an upregulation of Sox9 expression in response to stimulation with α-naphthoflavone, a competitive antagonist of the AhR. This might suggest that AhR is involved in the modulation of Sox9 activity, such as a constitutive inhibitory activity. 

The AhR has been shown to transactivate class I heterodimeric nuclear receptors while antagonising the activation of homodimeric ones in breast cancer cells [49]. The cAMP Response Element binding protein (CBP/p300) is an important coactivator of both the transcriptional activity of Sox9 and AhR (as reviewed in [50]). These data suggest a new uncharacterised interaction between the two transcription factors. Further experimental work will be necessary to validate this hypothesis. 

## 5. Conclusions

In summary, our data demonstrated a clear detrimental effect of HQ on articular cartilage homeostasis and shed novel insight into how environmental pollutants can exacerbate the degenerative effect of pro-inflammatory mechanisms underlying the onset of articular diseases. 

## Figures and Tables

**Table 1:** PCR primer sequences used for quantitative gene expression analysis.

Gene	Sense Primer	Anti-Sense Primer	Annealing Temperature (°C)
β-actin	AGCAGTCGGTTGGATCGAGCA	GGGAAGGCAAAGGACTTCCTGTAAC	55
SOX-9	GCTCTGGGCAAGCTCTGGAGACT	GGCGCGGCTGGTACTTGTAGTCC	60
Col2a1	ACGTCCAGATGACCTTCCTG	GGATGAGCAGAGCCTTCTTG	55
COL-X	AAAGGTCTAAGTGGCCCCTTTTGTC	GAGGTTCATGACAAAAGCACCTTGC	55
ADAMTS5	ACGGGACCGTCATGAACTAC	CTTTTGGAGCCGACTTCTTG	55
MMP-3	TGTGTGTCTTGCCCACTAGC	TGCCTGTTGCAGAATGCTAA	55
AhR	AGCAGCGCTAACATCACCTA	GGGACTGGCTTGACAGTTTTC	60
ARNT	CAGGTGCACCCAGATGATGT	AGATCCAGGATACGCCCTGT	60
Cyp1a1	ACCTTGATCACTAACGGCCA	GCCTCCTTGTTCACATGCTCT	60

## Data Availability

Not applicable.

## References

[1] Enguita F.J., Leitão A.L. (2013). Hydroquinone: Environmental pollution, toxicity, and microbial answers. Biomed Res. Int..

[2] Bodnar J.A., Morgan W.T., Murphy P.A., Ogden M.W. (2012). Mainstream smoke chemistry analysis of samples from the 2009 US cigarette market. Regul. Toxicol. Pharmacol..

[3] McGregor D. (2007). Hydroquinone: An evaluation of the human risks from its carcinogenic and mutagenic properties. Crit. Rev. Toxicol..

[4] Cho J.Y. (2008). Suppressive effect of hydroquinone, a benzene metabolite, on in vitro inflammatory responses mediated by macrophages, monocytes, and lymphocytes. Mediat. Inflamm..

[5] Lee J.S., Yang J.S., Yang E.J., Kim I.S. (2012). Hydroquinone-induced apoptosis of human lymphocytes through caspase 9/3 pathway. Mol. Biol. Rep..

[6] Li J., Jiang S., Chen Y., Ma R., Chen J., Qian S., Shi Y., Han Y., Zhang S., Yu K. (2018). Benzene metabolite hydroquinone induces apoptosis of bone marrow mononuclear cells through inhibition of β-catenin signaling. Toxicol. Vitr..

[7] Fogelholm R.R., Alho A.V. (2001). Smoking and intervertebral disc degeneration. Med. Hypotheses.

[8] Goldberg M.S., Scott S.C., Mayo N. (2000). A review of the association between cigarette smoking and the development of nonspecific back pain and related outcomes. Spine.

[9] Heluany C.S., Kupa L.V.K., Viana M.N., Fernandes C.M., Farsky S.H.P. (2018). Hydroquinone exposure worsens the symptomatology of rheumatoid arthritis. Chem. Biol. Interact..

[10] Heluany C.S., Kupa L.V.K., Viana M.N., Fernandes C.M., Silveira E.L.V., Farsky S.H.P. (2018). In vivo exposure to hydroquinone during the early phase of collagen-induced arthritis aggravates the disease. Toxicology.

[11] Heluany C., Donate P., Schneider A., Fabris A., Gomes R., Villas-Boas I., Tambourgi D., Silva T., Trossini G., Nalesso G. (2021). Hydroquinone Exposure Worsens Rheumatoid Arthritis through the Activation of the Aryl Hydrocarbon Receptor and Interleukin-17 Pathways. Antioxidants.

[12] Lin Z., Willers C., Xu J., Zheng M.-H. (2006). The chondrocyte: Biology and clinical application. Tissue Eng..

[13] Eyre D. (2001). Articular cartilage and changes in Arthritis: Collagen of articular cartilage. Arthritis Res. Ther..

[14] Sophia Fox A.J., Bedi A., Rodeo S.A. (2009). The basic science of articular cartilage: Structure, composition, and function. Sport. Health.

[15] Brand D.D., Latham K.A., Rosloniec E.F. (2007). Collagen-induced arthritis. Nat. Protoc..

[16] Nalesso G., Sherwood J., Bertrand J., Pap T., Ramachandran M., De Bari C., Pitzalis C., Dell’Accio F. (2011). WNT-3A modulates articular chondrocyte phenotype by activating both canonical and noncanonical pathways. J. Cell Biol..

[17] De Bari C., Dell’Accio F., Luyten F.P. (2001). Human periosteum-derived cells maintain phenotypic stability and chondrogenic potential throughout expansion regardless of donor age. Arthritis Rheum..

[18] Hayer S., Vervoordeldonk M.J., Denis M.C., Armaka M., Hoffmann M., Bäcklund J., Nandakumar K.S., Niederreiter B., Geka C., Fischer A. (2021). ‘SMASH’ recommendations for standardised microscopic arthritis scoring of histological sections from inflammatory arthritis animals models. Ann. Rheum. Dis..

[19] Farndale R.W., Sayers C.A., Barrett A. (1982). A direct spectrophotometric microassay for sulphated glycosaminoglycans in cartilage cultures. Connect. Tissue Res..

[20] Firestein G.S. (2003). Evolving concepts of rheumatoid arthritis. Nature.

[21] Dell’Accio F., De Bari C., Luyten F.P. (2001). Molecular markers predictive of the capacity of expanded human articular chondrocytes to form stable cartilage in vivo. Arthritis Rheum..

[22] Sandell L.J., Aigner T. (2001). Articular cartilage and changes in arthritis. An introduction: Cell biology of osteoarthritis. Arthritis Res..

[23] Mao J., Dai W., Zhang S., Sun L., Wang H., Gao Y., Wang J., Zhang F. (2019). Quinone-thioether metabolites of hydroquinone play a dual role in promoting a vicious cycle of ROS generation: In vitro and in silico insights. Arch. Toxicol..

[24] Henrotin Y.E., Bruckner P., Pujol J.P. (2003). The role of reactive oxygen species in homeostasis and degradation of cartilage. Osteoarthr. Cartil..

[25] Amin A., Abramson S.B. (1998). The role of nitric oxide in articular cartilage breakdown in osteoarthritis. Curr. Opin. Rheumatol..

[26] Daheshia M., Yao J.Q. (2008). The interleukin 1beta pathway in the pathogenesis of osteoarthritis. J. Rheumatol..

[27] Jenei-Lanzl Z., Meurer A., Zaucke F. (2019). Interleukin-1β signalling in osteoarthritis—Chondrocytes in focus. Cell. Sig..

[28] Sellan J., Berenbaum F. (2010). The role of synovitis in pathophysiology and clinical symptoms of osteoarthritis. Nat. Rev. Rheumatol..

[29] Ma Q., Baldwin K.T. (2000). 2,3,7,8-tetrachorodibenzo-p-dioxin- induced degradation of aryl hydrocarbon receptor (AhR) by the ubiquitin-proteasome pathway. Role of the transcription activation and DNA binding. J. Biol. Chem..

[30] Larigot L., Juricek L., Dairou J., Coumoul X. (2018). AhR signaling pathways and regulatory functions. Biochim. Open.

[31] Finckh A., Gilbert B., Hodkinson B., Bae S.-C., Thomas R., Deane K.D., Alpizar-Rodriguez D., Lauper K. (2022). Global epidemiology of rheumatoid arthritis. Nat. Rev. Rheumatol..

[32] Cui A., Li H., Wang D., Zhong L., Chen Y., Lu H. (2020). Global, regional prevalence, incidence and risk factors of knee osteoarthritis in population-based studies. EClinicalMedicine.

[33] Mort J.S., Billinton C.J. (2001). Articular cartilage and changes in arthritis matrix degradation. Arthritis Res..

[34] Goldring M.B., Goldring S.R. (2010). Articular cartilage and subchondral bone in the pathogenesis of osteoarthritis. Ann. N. Y. Acad. Sci..

[35] Arner E.C. (2002). Aggrecanase-mediated cartilage degradation. Curr. Opin. Pharmacol..

[36] Poole A.R., Kobayashi M., Yasuda T., Laverty S., Mwale F., Kojima T., Sakai T., Wahl C., El-Maadawy S., Webb G. (2001). Type II collagen degradation and its regulation in articular cartilage in osteoarthritis. Ann. Rheum. Dis..

[37] Bortoluzzi A., Furini F., Sciré C.A. (2018). Osteoarthritis and its management—Epidemiology, nutritional aspects and environmental factors. Autoimmun. Rev..

[38] Suantawee T., Tantavisut S., Adisakwattana S., Tanavalee A., Yuktanandana P., Anomasiri W., Deepaisarnsakul B., Honsawek S. (2013). Oxidative stress, vitamin e, and antioxidant capacity in knee osteoarthritis. J. Clin. Diagn. Res..

[39] Abramson S.B. (2008). Osteoarthritis and nitric oxide. Osteoarthr. Cartil..

[40] Lee H.G., Yang J.H. (2012). PCB126 induces apoptosis of chondrocytes via ROS-dependent pathways. Osteoarthr. Cartil..

[41] Pons M., Cousins S.W., Csaky K.G., Striker G., Marin-Castaño M.E. (2010). Cigarette smoke-related hydroquinone induces filamentous actin reorganization and heat shock protein 27 phosphorylation through p38 and extracellular signal-regulated kinase 1/2 in retinal pigment epithelium: Implication for age-related macular degeneration. Am. J. Pathol..

[42] Peng C., Arthur D., Liu F., Lee J., Xia Q., Lavin M.F., Ng J.C. (2013). Genotoxicity of hydroquinone in A549 cells. Cell Biol. Toxicol..

[43] Julliard W., Fechner J.H., Mezrich J.D. (2014). The aryl hydrocarbon receptor meets immunology: Friend or foe? A little of both. Front. Immunol..

[44] Denison M.S., Nagy S.R. (2003). Activation of the aryl hydrocarbon receptor by structurally diverse exogenous and endogeneous chemicals. Ann. Rev. Pharmacol. Toxicol..

[45] Nakahama T., Kimura A., Nguyen N.T., Chinen I., Hanieh H., Nohara K., Fujii-Kuriyama Y., Kishimoto T. (2011). Aryl hydrocarbon receptor deficiency in T cells suppresses the development of collagen-induced arthritis. Proc. Natl. Acad. Sci. USA.

[46] Nguyen N.T., Nakahama T., Nguyen C.H., Tran T.T., Le V.S., Chu H.H., Kishimoto T. (2015). Aryl hydrocarbon receptor antagonism and its role in rheumatoid arthritis. J. Exp. Pharmacol..

[47] Kobayashi S., Okamoto H., Iwamoto T., Toyama Y., Tomatsu T., Yamanaka H., Momohara S. (2008). A role for the aryl hydrocarbon receptor and the dioxin TCDD in rheumatoid arthritis. Rheumatology.

[48] Talbot J., Peres R.S., Pinto L.G., Oliveira R.D.R., Lima K.A., Donate P.B., Silva J.R., Ryffel B., Cunha T.M., Alves-Filho J.C. (2018). Smoking-induced aggravation of experimental arthritis is dependent of aryl hydrocarbon receptor activation in Th17 cells. Arthritis Res. Ther..

[49] Widerak M., Ghoneim C., Dumontier M.-F., Quesne M., Corvol M.T., Savouret J.-F. (2006). The aryl hydrocarbon receptor activates the retinoic acid receptor α through SMRT antagonism. Biochimie.

[50] Gargaro M., Scalisi G., Manni G., Mondanelli G., Grohmann U., Fallarino F. (2021). The Landscape of AhR regulators and coregulators to fine-tune AhR functions. Int. J. Mol. Sci..

